# Insights into the Effect of Aggregate Sizes on the Soil Radiation Interaction Properties Based on X-ray Fluorescence

**DOI:** 10.3390/ijerph192214635

**Published:** 2022-11-08

**Authors:** Lohane Tech, Luiz F. Pires

**Affiliations:** 1 Physics Graduate Program, State University of Ponta Grossa, Ponta Grossa 84030-090, Brazil; 2 Department of Physics, State University of Ponta Grossa, Ponta Grossa 84030-090, Brazil

**Keywords:** effective atomic number, electronic cross-section, mass attenuation coefficient, soil chemical composition, soil granulometric fractions, soil microaggregates

## Abstract

Soils subjected to disaggregation can break into aggregates of different sizes composed of sand, clay, and silt particles. Each aggregate contains different oxides, which can vary according to the aggregate size and influence its properties, such as the radiation interaction parameters. These parameters are relevant in the evaluation of radiation shielding and soil physical properties. Thirteen tropical/subtropical soils of contrasting textures (clayey and loamy/sandy) with two aggregate sizes (2–1 mm and <45 μm) were studied. The radiation parameters analyzed were the atomic (σ_A_), electronic (σ_E_), and molecular (σ_M_) cross-sections; the effective atomic number (Z_eff_); and the electron density (N_el_). We verified that the aggregate sizes affected the major oxides (SiO_2_, Al_2_O_3_, Fe_2_O_3_). In general, the attenuation coefficient and Z_eff_ were sensitive to the clayey soils’ aggregate sizes (low photon energies). However, the loamy/sandy soils did not exhibit differences among the parameters. As the photon energy increased, only Z_eff_ presented differences for most soils. We also verified that σ_M_, Z_eff_, and N_el_ were the most sensitive parameters to the soil composition. Although the soil chemical composition was influenced by the studied aggregate sizes, the radiation parameters exhibited differences for only some of these parameters. This means that the aggregate size is practically irrelevant when radiation parameters are determined based on X-ray fluorescence.

## 1. Introduction

When photons interact with different composite materials, many processes (photoelectric effect, coherent and incoherent scattering, and pair production) can occur, which results in their absorption or scattering [1]. The processes of radiation interaction with the soil allow the investigation of different physical properties of this porous media, such as bulk density, porosity, water retention, and hydraulic conductivity [2,3,4]. Attenuation coefficient measurements enable the determination of many physical parameters of interest to the radiation shielding field [5,6].

Soils contain particles of different sizes. Soil granulometry analysis allows its characterization in terms of its clay, silt, and sand contents [7]. Different proportions of these particles make it possible to classify the soil into distinct types. Sand particles present sizes between 2,000 and 50 μm, silt between 50 and 2 μm, and clay <2 μm. Thus, when photons interact with soil samples, this interaction occurs with different aggregate sizes and particles. The clay fraction comprises mineral particles known as aluminosilicates (kaolinite, illite, goethite, among others). The silt fraction has quartz and minerals such as kaolinite and mica, while the sand fraction is mainly composed of quartz [8]. This means that soils present a complex composition regarding chemical elements (oxides), affecting how radiation interacts with this porous medium [9,10].

In tropical/subtropical soils, iron (Fe_2_O_3_) and aluminum oxides (Al_2_O_3_) are essential materials acting in their structure stabilization [11]. The structure stability will contribute to the soil aggregate stability, meaning that the soil can become either more or less resistant to disaggregate forces [12]. Under disaggregation, the soil can be broken into different aggregate sizes presenting distinct chemical compositions, which might influence the radiation interaction parameters [13].

One of the most important parameters to measure the radiation interaction with soils is the mass attenuation coefficient (μ/ρ). It can be measured experimentally or through computational methods [14,15]. The latter are usually based on previous analysis of the soil chemical composition employing X-ray fluorescence (XRF) techniques [2,16,17,18]. The knowledge of μ/ρ allows the calculation of other essential interaction parameters for the radiation physics field, such as the atomic (σ_A_), electronic (σ_E_), and molecular (σ_M_) cross-sections; the effective atomic number (Z_eff_); and the electron density (N_el_) [19,20,21].

Therefore, knowing how photons with different energies interact with soil aggregates might provide highly relevant information regarding how radiation interaction processes occur in this porous medium. Thus, it is possible to verify whether soils composed of different aggregate sizes can be either more or less effective in attenuating radiation. Consequently, parameters such as the half-layer thickness (not covered in this paper) might depend on how the soil is aggregated [22]. Furthermore, it is important to mention that soil physical properties, such as the bulk density, porosity, and water content, are based on knowledge of the attenuation coefficient when measured through nuclear methods (e.g., gamma-ray attenuation) [2,3,9,10]. Therefore, if μ/ρ depends on the aggregate sizes, its value might influence the evaluation of these properties.

It is also worth mentioning that soil is an abundant material, easily found in nature and cheap, with potential to be used in the field of radiation shielding [5,6]. Thus, studying materials to shield against radiation has great environmental appeal in the use of radioactive sources for energy production, medical applications, space and military purposes, etc. However, these radiation sources can generate significant amounts of waste, possibly exposing people to the effects of ionizing radiation when they are inappropriately managed [6]. Therefore, employing alternative materials such as soil and its fractions for radiation shielding is an interesting and cost-effective alternative. Furthermore, considering that soils present varied types of structures comprising different sizes of aggregates [7], a detailed analysis of how radiation will interact with them is of great interest.

Taking this into consideration, this study proposes a detailed analysis of how two aggregate sizes (2–1 mm and <45 μm) might affect the soil chemical composition measurement by XRF and consequently influence the radiation interaction parameters. No previous study has evaluated how soil aggregate sizes influence the radiation interaction parameters. Additionally, no other studies have verified the existence of optimum aggregate sizes for XRF analysis, presenting detailed information about radiation interaction parameters in tropical/subtropical soils of different chemical compositions for contrasting aggregate sizes. Therefore, our study aimed to propose some answers to these questions.

## 2. Materials and Methods

Thirteen soils of varied textures were collected in different experimental stations located in the Paraná State (22°30′58″ S–26°43′00″ S and 48°05′37″ W–54°37′08″ W), south of Brazil. Data about each soil’s clay, silt, and sand amounts are shown in Table 1. The thirteen soils were divided into two groups (clay and loam/sand) to facilitate the analysis of the results. Their textural classes were considered to separate the soils into groups (Figure S1—Supplementary Materials). The granulometry analysis of the soils was carried out at the Paraná Agronomic Institute (Ponta Grossa–PR) using the pipette method, which is based on the fall velocity of the soil particles in a dispersant solution (NaOH) [23]. The sand fraction was separated by sieving. Next, the fine soil fractions (silt and clay) were separated by decantation. Stoke’s law was used to estimate the sedimentation time for the silt particles (c. 8 h). After this time, a suspension volume was pipetted and dried in an oven, corresponding to the clay fraction. Silt was determined by the difference between the other two fractions [24].

All the soil samples were previously dried in air and sieved in a sieve mesh with a size of 2–1 mm since the aggregate analysis required this diameter interval. After sieving, samples were kept in an oven at 45 °C (air forced circulation) to avoid air humidity. Next, following the 2–1 mm aggregate analysis, the same samples were sieved in a 45 μm wire-mesh sieve to study the small aggregate sizes. It is worth mentioning that our laboratory utilizes aggregates of <45 μm size for routine XRF analysis. The technique employed in the elementary analysis was energy-dispersive X-ray fluorescence. The measurements were carried out at the Laboratory of XRF Analysis (Department of Physics, State University of Ponta Grossa). The equipment (Shimadzu, model EDX-720) has a rhodium tube (Rh), its voltage varies from 5 to 50 kV, and the filament operates with currents between 1 and 1000 μA. The detector is a Si(Li) semiconductor cooled by liquid nitrogen at −196 °C. For the XRF analyses, soil samples were mashed in pestles and placed on sample holders (c. 2 g) provided by the equipment manufacturer (c. 22 mm diameter), which were sealed with mylar (6 μm thickness). A time interval of about 100 s was set to obtain each spectrum (200 s total time) in the energy bands of sodium to scandium (Na–Sc) (5 kV) and titanium to uranium (Ti–U) (50 kV). All the measurements were carried out in the semi-quantitative mode under a 30 Pa pressure (vacuum) [16,25].

Three measurements (n = 3) were obtained for all parameters analyzed (μ/ρ, σ_A_, σ_E_, σ_M_, Z_eff_, and N_el_). The soil chemical compositions were employed to calculate the mass attenuation coefficients via the XCOM program [26]. This computational code generates cross-sections for the photoelectric effect, coherent and incoherent scatterings, and pair production [26]. The combination of the partial cross-sections allows the determination of total μ/ρ for any element, compound, and mixture in the photon energy range from 1 keV to 100 GeV [27].

The parameters μ/ρ, σ_A_, σ_E_, σ_M_, Z_eff_, and N_el_ were calculated based on the soil chemical compositions. We utilized the equations shown below for the calculation of the mentioned radiation interaction parameters.

To calculate Z_eff_ and N_el_ of compounds and mixtures, we need to know the molecular, atomic, and electronic cross-sections. Thus, we used the following equation to calculate the molecular cross-section:

(1) $$\sigma_{M}=\left(\frac{\mu }{\rho }\right)\frac{M}{N_{A}},$$

where μ⁄ρ is the mass attenuation coefficient, $M=\underset{i}{\sum }n_{i}A_{i}$ is the molecular weight of the compound, N_A_ is the Avogadro number, A_i_ is the atomic weight of the i-th element, and n_i_ is the number of formula units in the molecule.

The average atomic cross-section is obtained by dividing σ_M_ by the total number of formula units as follows:

(2) $$\sigma_{A}=\sigma_{M}\frac{1}{\sum_{i}n_{i}},$$

where $\underset{i}{\sum }n_{i}$ is the total number of formula units of the compound.

To obtain the electronic cross-section, we used the following equation:

(3) $$\sigma_{E}=\frac{1}{N_{A}}\underset{i}{\sum }f_{i}\frac{A_{i}}{Z_{i}}{\left(\frac{\mu }{\rho }\right)}_{i},$$

where $f_{i}=\frac{n_{i}}{\sum_{j}n_{j}}$ and Z_i_ are the fractional abundance and the atomic number of the i-th constituent element, with n_j_ being the number of atoms of the constituent element and $\underset{j}{\sum }n_{j}$ being the total number of atoms present in the molecular formula.

Combining Equations (2) and (3), we obtained the effective atomic number:

(4) $$Z_{\mathrm{eff}}=\frac{\sigma_{A}}{\sigma_{E}},$$

The electron density was calculated using:

(5) $$N_{\mathrm{el}}=\frac{\left(\mu /\rho \right)}{\sigma_{E}},$$

This study investigated the energy interval from 1 keV to 100 MeV. However, we emphasized two photon energies: c. 60 and c. 662 keV, corresponding to the gamma-rays emitted by the ^241^Am and ^137^Cs radioactive sources. We selected these two energies due to their broad utilization in experimental measurements of soil physical properties by gamma-ray attenuation and gamma-ray tomography [19,22,27,28].

Statistical analysis was performed using the PAleontological STatistics (PAST) software version 3.21 (Oslo, Norway). Comparisons between aggregate sizes and soils (all parameters analyzed) were carried out based on the mean standard deviation. In addition, Pearson correlations among each pair of variables were performed for all the parameters. This analysis considered all the aggregate sizes and soils, aiming to show possible general trends among the variables analyzed.

## 3. Results

The percentages of the most common oxides found in the 13 soils studied and 2 aggregate sizes (2–1 mm and <45 μm) considering both textural groups (clay and loam/sand) are presented in the sequence (Table 2).

**Figure 1:**
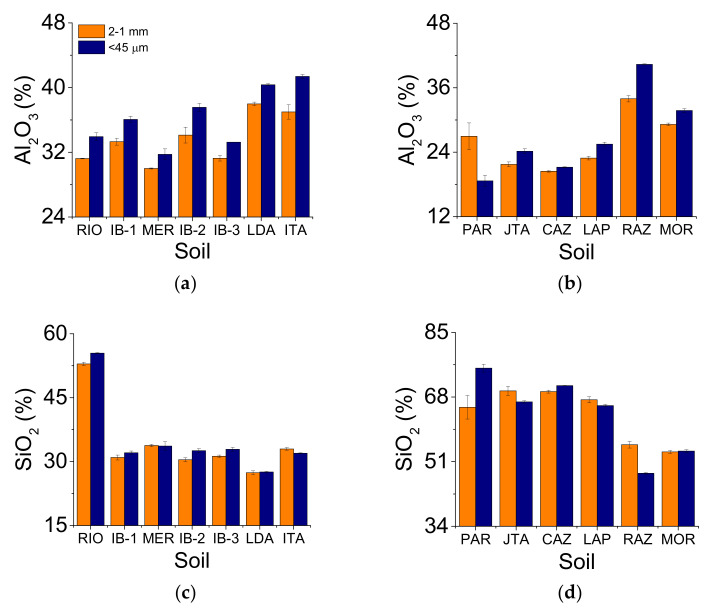

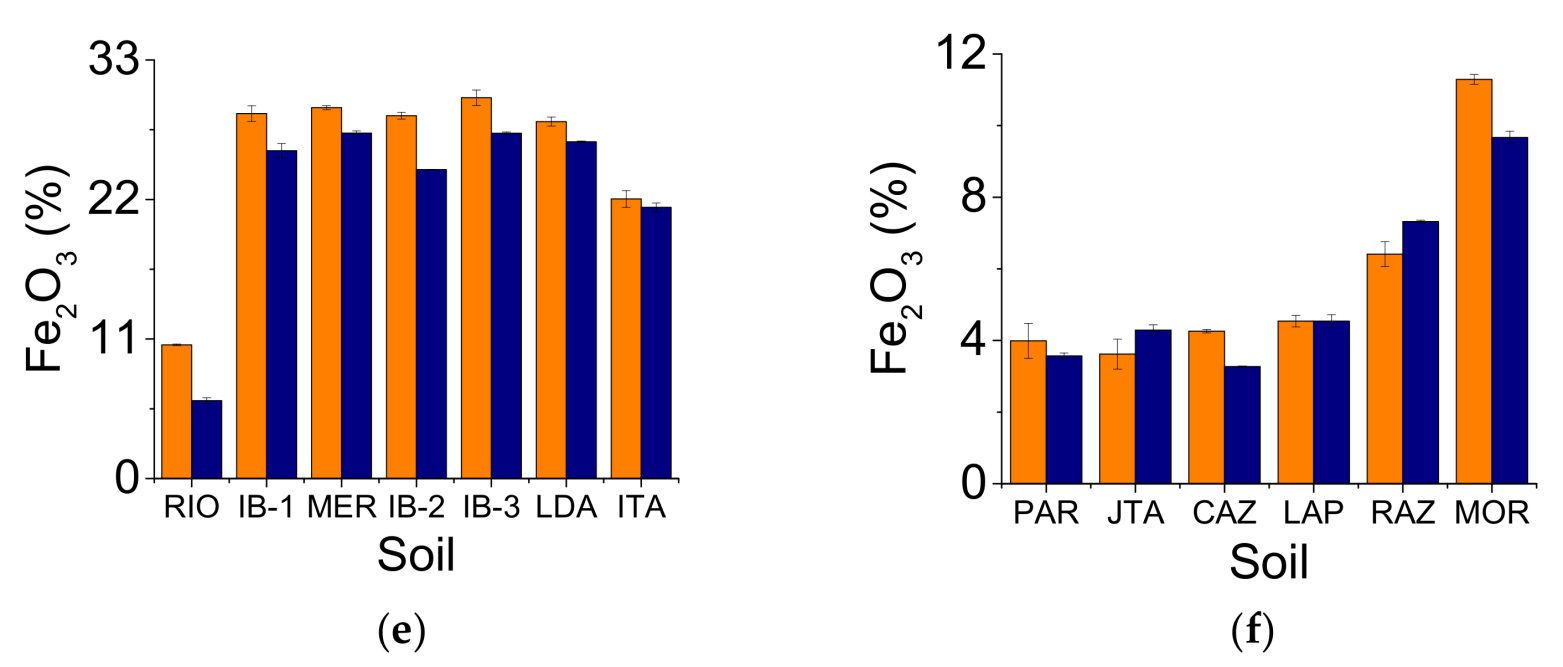
(**a**,**c**,**e**) Major soil oxide contents (Al_2_O_3_, SiO_2_, Fe_2_O_3_) for the different aggregate sizes (2–1 mm and <45 μm) for the clayey soils. (**b**,**d**,**f**) Major soil oxide contents for the different aggregate sizes for the loamy/sandy soils. Vertical bars represent the standard deviation (n = 3).

In the clayey soils, the most abundant oxides were Al_2_O_3_ and SiO_2_ (Figure 1a,c), with other oxides showing the following sequence (most of the soils): Fe_2_O_3_ > TiO_2_ > SO_3_ (Tables S1 and S2—Supplementary Materials). In the loamy/sandy soils, the most abundant oxide was SiO_2_ (Figure 1d) followed by: Al_2_O_3_ > Fe_2_O_3_ > TiO_2_ > SO_3_ (Tables S1 and S2—Supplementary Materials). No direct relation was observed between the clay content and the amount of iron oxide in the clayey soils analyzed. A strong positive correlation between clay × Al_2_O_3_ (r = 0.73, *p* = 0.06) and sand × SiO_2_ (r = 0.83, *p* < 0.05) was found. However, for the loamy/sandy soils, with the exception of CAZ, which was not considered in the correlation analysis due to its high silt content, positive relations between clay × Al_2_O_3_ (r = 0.89, *p* < 0.05), clay × Fe_2_O_3_ (r = 0.90, *p* < 0.05), silt × Al_2_O_3_ (r = 0.93, *p* < 0.05), and sand × SiO_2_ (r = 0.97, *p* < 0.05) were observed. On the other hand, negative relations were found between sand × Al_2_O_3_ (r = −0.93, *p* < 0.05), clay × SiO_2_ (r = −0.95, *p* < 0.05), and silt × SiO_2_ (r = −0.95, *p*< 0.05). We must mention that these correlation analyses were carried out for the <45 μm aggregate size.

When the oxide percentages in the 2–1 mm and <45 μm aggregate sizes were compared, the Al_2_O_3_ concentration increased in the latter in the clayey soils (Table 1 and Table 2 and Figure 1a). Regarding SiO_2_ (Figure 1c), differences were verified between the aggregate sizes for RIO, IB-2, IB-3, and ITA (Table 2). For Fe_2_O_3_ (Figure 1e), the 2–1 mm aggregate size exhibited the highest concentration among the clayey soils (Table 2). However, even with the differences observed in the major soil oxides (Al_2_O_3_, SiO_2_, Fe_2_O_3_) among the clayey soils, we verified that most of them presented the same variation trend, regardless of the aggregate size. In the loamy/sandy soils, small aggregates (<45 μm) presented the highest concentration of Al_2_O_3_ (Figure 1b), except for PAR (Table 2). For SiO_2_ (Figure 1d), the 2–1 mm aggregates exhibited, in general, the highest concentrations among the soils, except for PAR and CAZ. MOR was a unique soil with no differences between the aggregate sizes for SiO_2_ (Table 2). For Fe_2_O_3_ (Figure 1f), we did not find a clear trend in its concentration among the aggregate sizes. Soils with differences between the aggregate sizes for Fe_2_O_3_ were JTA, CAZ, RAZ, and MOR (Table 2). However, we noticed that for the loamy/sandy soils, only Al_2_O_3_ demonstrated a similar trend between the aggregate sizes.

The mass attenuation coefficients calculated for the specific photon energies (c. 60 and c. 662 keV) and both aggregate sizes (2–1 mm and <45 μm) of the contrasting soils studied are presented in the sequence (Table 3). We also compared the calculated results with the experimental results, which showed good agreement between the methods. The relative changes ranged from 0.8% to 6.4% for the clayey soils and from 0.5% to 7.2% for the loamy/sandy soils (Table S3—Supplementary Materials).

When the aggregate size of the clayey soils decreased, μ/ρ increased for the low energy photons (c. 60 keV) (Table 3). However, no difference was noticed between the aggregate sizes in RIO, MER, IB-2, and IB-3. Nonetheless, the μ/ρ variation trend for the different aggregate sizes (2–1 mm and <45 μm) was similar. The correlation analyses among the different soil oxides, granulometric composition, and μ/ρ showed strong to moderate linear relations between Fe_2_O_3_ × μ/ρ (r = 0.99, *p* < 0.05), SiO_2_ × μ/ρ (r = −0.92, *p* <0.05), and sand × μ/ρ (r = −0.61, *p* = 0.14) for the 2–1 mm aggregates. Regarding the small aggregates (<45 μm), we found a strong linear relation between Fe_2_O_3_ × μ/ρ (r = 0.99, *p* = 0.14), SiO_2_ × μ/ρ (r = −0.95, *p* < 0.05), and sand × μ/ρ (r = −0.74, *p* = 0.06). Correlation analysis was carried out for the largest aggregates considering the oxide content determined for this specific aggregate size and the same procedure was carried out for the smallest aggregates. No differences were found among the clayey soils for the high energy (c. 662 keV) (Table 3).

For the loamy/sandy soils (Table 3), we did not find differences in μ/ρ between the aggregate sizes (2–1 mm and <45 μm) for the low energy (c. 60 keV). We practically verified the same μ/ρ variation trend among the soils for the different aggregate sizes, except for JTA, LAP, and RAZ. The correlation analysis showed moderate to strong linear relations between SiO_2_ × μ/ρ (r = −0.68, *p* = 0.14), Fe_2_O_3_ × μ/ρ (r = 0.95, *p* < 0.05), and clay × μ/ρ (r = 0.73, *p* = 0.10) for the 2–1 mm aggregate sizes. For the small aggregates (<45 μm), we observed moderate to strong linear relations between Fe_2_O_3_ × μ/ρ (r = 0.83, *p* < 0.05) and clay × μ/ρ (r = 0.68, *p* = 0.06), similar to the results of the largest aggregates. For the high energy (c. 662 keV), we noticed practically the same μ/ρ variation trend among the aggregate sizes (Table 3).

**Figure 2:**
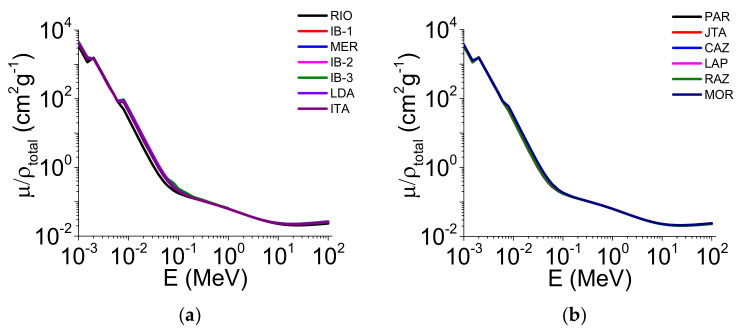

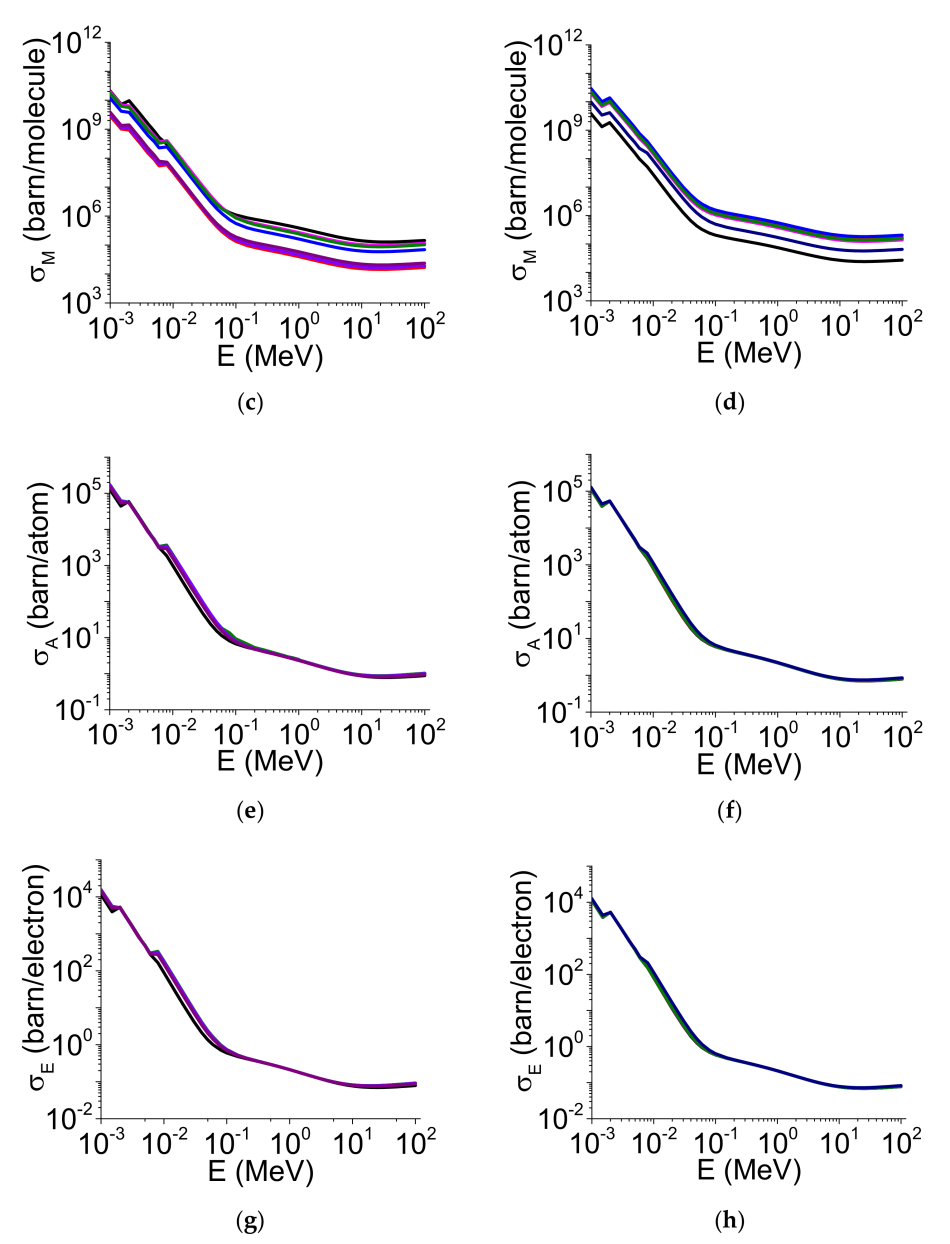
Mass attenuation coefficient (μ/ρ), molecular cross-section (σ_M_), atomic cross-section (σ_A_), and electronic cross-section (σ_E_) variation as a function of the photon energy (E) (1 keV–100 MeV) for the 2–1 mm aggregate sizes in the clayey soils (**a**,**c**,**e**,**g**) and the loamy/sandy soils (**b**,**d**,**f**,**h**).

**Figure 3:**
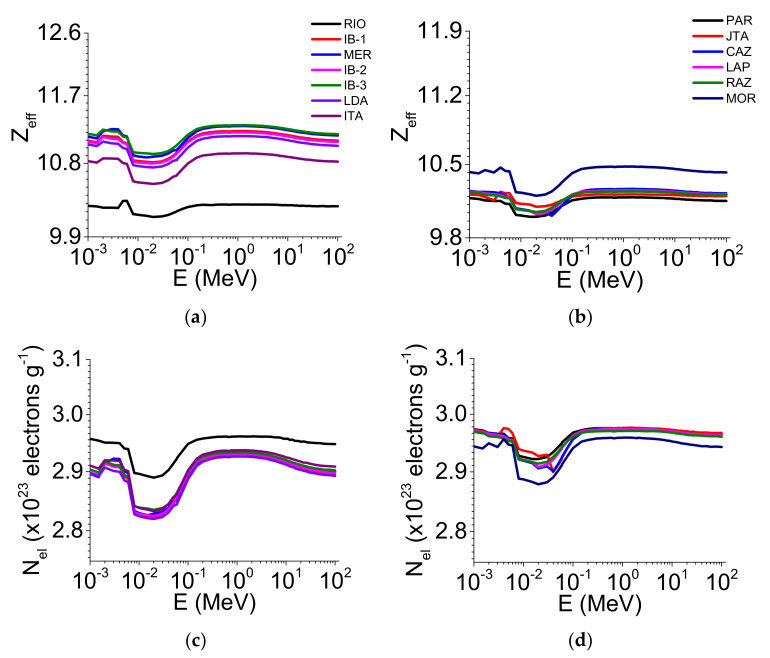
Effective atomic number (Z_eff_) and electron density (N_el_) variation as a function of the photon energy (E) (1 keV–100 MeV) for the 2–1 mm aggregate sizes in the clayey soils (**a**,**c**) and the loamy/sandy soils (**b**,**d**).

Figure 2 and Figure 3 show the μ/ρ, σ_M_, σ_A_, σ_E_, Z_eff_, and N_el_ variations as a function of the photon energy for the 2–1 mm aggregate sizes, considering the 2 soil groups studied (clay and loam/sand).

The mass attenuation coefficient (Figure 2a,b) presented a sharp decrease between 10^−3^ and ~10^−1^ MeV photon energies in all the soils investigated. In the intermediate to high energy region >10 MeV, a small increase in μ/ρ was noticed. We found that only some clayey soils (RIO, IB-1, LDA, ITA) showed differences in μ/ρ (Figure 2a), which occurred in the energy region from ~10^−2^ to ~10^−1^ MeV (Table 3). The molecular cross-section (Figure 2c,d) was more sensitive to the soil chemical composition than μ/ρ. Among the clayey soils, the lowest σ_M_ was found in IB-1 (10^−3^ to ~10^2^ MeV) while RIO (E < 10^−2^ MeV and E > 10^−1^ MeV) presented the highest value (Figure 2c). For the energy region 10^−2^ < E < 10^−1^ MeV, IB-2 showed the highest σ_M_ (Figure 2c). The same behavior was observed in the loamy/sandy soils (Figure 2d), with the lowest value observed in PAR and the highest in CAZ (10^−3^ to 10^2^ MeV). The parameters σ_A_ and σ_E_ (Figure 2e–h) showed similar μ/ρ behavior in both soil groups. The highest differences among the soils investigated were slight and found for the clayey soils in the energy region of ~10^−2^ to ~10^−1^ MeV (Figure 2e,g).

The Z_eff_ (Figure 3a,b) and N_el_ parameters (Figure 3c,d) were also influenced by the soil chemical composition, as noticed for σ_M_. RIO exhibited the lowest Z_eff_ (Figure 3a) while IB-3 and MER showed the highest values (10^−3^ to ~10^2^ MeV) in the clayey soils. For the loamy/sandy soils, PAR and CAZ had the lowest Z_eff_ (Figure 3b) and MOR the highest (10^−3^ to ~10^2^ MeV). Regarding N_el_, its lowest and highest values occurred in LDA and RIO, respectively, for the clayey soils (Figure 3c). In contrast, MOR presented the lowest value in the loamy/sandy soils while PAR and JTA showed the highest values in the energy region from 10^−3^ to ~10^2^ MeV (Figure 3d).

**Figure 4:**
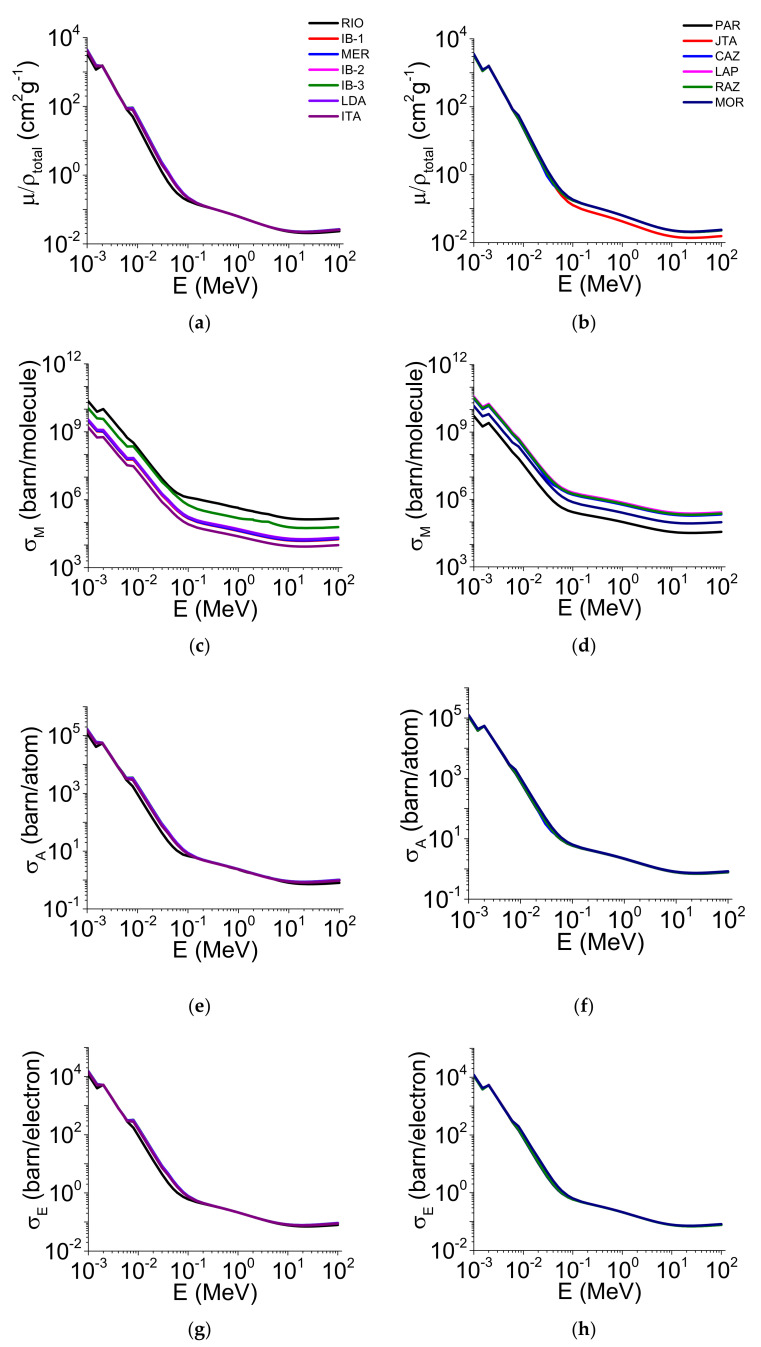
Mass attenuation coefficient (μ/ρ), molecular cross-section (σ_M_), atomic cross-section (σ_A_), and electronic cross-section (σ_E_) variation as a function of the photon energy (E) (1 keV–100 MeV) for the <45 μm aggregate sizes in the clayey soils (**a**,**c**,**e**,**g**) and the loamy/sandy soils (**b**,**d**,**f**,**h**).

**Figure 5:**
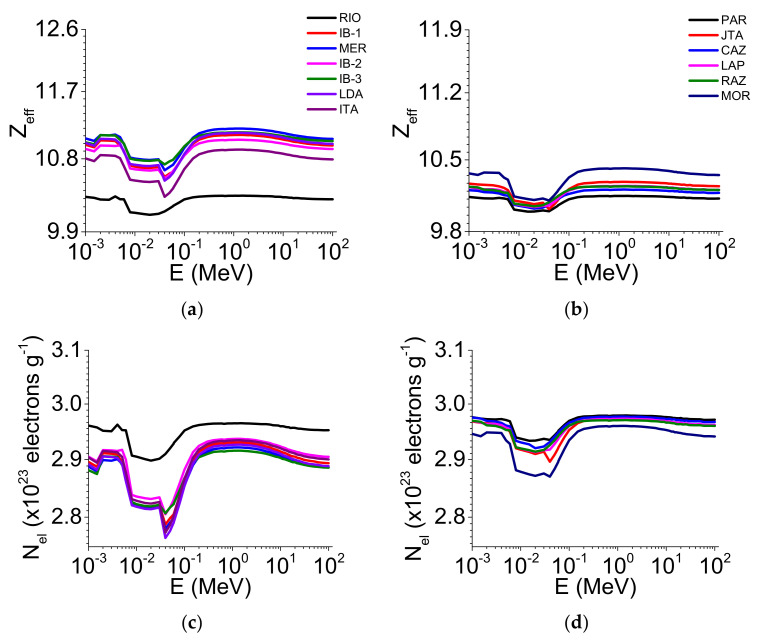
Effective atomic number (Z_eff_) and electron density (N_el_) variation as a function of the photon energy (E) (1 keV–100 MeV) for the <45 μm aggregate sizes in the clayey soils (**a**,**c**) and the loamy/sandy soils (**b**,**d**).

The results of the μ/ρ, σ_M_, σ_A_, σ_E_, Z_eff_, and N_el_ variation as a function of the photon energy for the <45 μm aggregate sizes for both soil groups (clay and loam/sand) are presented in Figure 4 and Figure 5.

When comparing the two aggregate sizes, we observed that only some soils’ μ/ρ was influenced by the different sizes. For the clayey soils, μ/ρ presented variation between the aggregate sizes in the energy region of ~10^−2^ to ~10^1^ MeV. For ~10^−2^ MeV, most of the 2–1 mm aggregate sizes showed the highest μ/ρ when compared with <45 μm. However, for ~10^−3^ MeV, the aggregates <45 μm showed the highest values of μ/ρ (Figure 2a and Figure 4a). For the loamy/sandy soils (Figure 2b and Figure 4b), only JTA presented differences for the smallest aggregate size (E > 10^−1^ MeV). For σ_M_ (Figure 2c and Figure 4c), considering the energies of c. 60 and c. 662 keV (Tables S4 and S5—Supplementary Materials), MER, IB-2, and ITA (clayey soils) showed the highest values for the 2–1 mm aggregate sizes (c. 60 keV). The same behavior was observed for c. 662 keV, with MER, IB-2, and ITA showing differences for the 2–1 mm aggregate sizes when compared to <45 μm (Tables S4 and S5—Supplementary Materials). For the loamy/sandy soils (Figure 2d and Figure 4d), the <45 μm aggregate sizes presented the highest σ_M_ only in LAP and MOR (c. 60 and c. 662 keV) (Tables S4 and S5—Supplementary Materials). The σ_A_ and σ_E_ parameters showed slight differences among the clayey soils (Figure 2e,g and Figure 4e,g) for c. 60 and c. 662 keV (Tables S4 and S5—Supplementary Materials). A similar behavior was observed among the loamy/sandy soils (Figure 2f,h and Figure 4f,h) for both energies (Tables S4 and S5—Supplementary Materials). For Z_eff_ and N_el_, the 2–1 mm aggregate sizes presented the highest values for most clayey soils (Figure 3a,c and Figure 5a,c) for c. 60 and c. 662 keV (Tables S4 and S5—Supplementary Materials). For the loamy/sandy soils (Figure 3b,d and Figure 5b,d), Z_eff_ and N_el_ were almost the same among the soils for c. 60 keV, except for MOR (Z_eff_). For c. 662 keV, only some soils showed differences (PAR, JTA, CAZ, MOR—Z_eff_ and JAT, CAZ, LAP—N_el_) (Tables S4 and S5—Supplementary Materials).

## 4. Discussion

This paper presents a detailed analysis of the effects of two aggregate sizes (2–1 mm and <45 μm) on the soil chemical compositions and radiation interaction parameters. The aggregate sizes influenced the composition of the major oxides (SiO_2_, Al_2_O_3_, Fe_2_O_3_) found in the clayey soils (Table 2 and Figure 1). This finding is mainly related to the role of aluminum and iron oxides in soil aggregation [12]. These two oxides and organic matter (not covered in this study) are important cement agents [13,29]. In tropical/subtropical soils, aluminum and iron oxides cover the soil particles and produce clay mineral layers, increasing the soil aggregate stability [30]. In clayey soils, the largest aggregates showed the highest Fe_2_O_3_ content while the production of microaggregates smaller than 45 μm reduced its concentration, favoring the increased SiO_2_ and Al_2_O_3_ concentrations. In the loamy/sandy soils, aluminum oxide presented the highest concentration for the smallest aggregate sizes, similar to the clayey soils. This result confirms the influence of this oxide as a cementing agent (Table 2). Silicon and iron oxides did not present any trend between the soil groups studied for the contrasting aggregate sizes.

Despite the differences in the soil chemical compositions, the mass attenuation coefficient did not present significant differences (c. 60 keV) in both soil groups (majority of clayey soils) and aggregate sizes (Table 3). Even the differences observed in the high Z oxides, which generally influence μ/ρ for the lowest photon energies, did not promote significant differences between the aggregate sizes among the soils (majority of clayey soils). This means that the balance between the other major oxides is also vital to μ/ρ [31,32]. However, we noticed that the Fe_2_O_3_ oxide influenced μ/ρ while the increase in the SiO_2_ content decreased μ/ρ, as observed for both aggregate sizes (clay and loam/sand) [9,15,19,33,34]. The increase in the photon energy (c. 662 keV) did not cause differences in μ/ρ between the aggregate sizes for the clayey and loamy/sandy soils. The characteristics of the samples analyzed (low density, similar oxide contents, particle sizes, etc.) can help to explain the results of μ/ρ for both photon energies [2,10,15,35,36].

The mass attenuation coefficient (Figure 2a,b and Figure 4a,b) presented a sharp decrease with the increase in the photon energy for the different aggregate sizes and soil groups [9,36,37]. In the low photon energy region (10^−3^ to ~10^−1^ MeV), μ/ρ was influenced mainly by the photoelectric effect, whose cross-section is proportional to Z^4−5^ and inversely proportional to the photon energy (E^−3^ when <0.5 MeV) [1,9]. For the intermediate energy region (~10^−1^ to ~10^1^ MeV), the decrease in μ/ρ was less abrupt due to the Compton effect (incoherent scattering) and cross-section Z dependence. In this energy band, the incoherent scattering is the dominant effect of the radiation attenuation. For the high energy region (>10 MeV), μ/ρ presented a slight increase influenced by the pair production, whose cross-section shows dependency with Z^2^ [38,39].

The molecular cross-section (Figure 2c,d and Figure 4c,d) was greatly influenced by the molecular weight of the soil constituents (Equation (1)). The differences in the soil chemical compositions might explain the verified σ_M_ distinctions among the studied soils (both soil groups and aggregate sizes) [16]. For c. 60 keV, some clayey soils presented the highest σ_M_ for the 2–1 mm aggregates while the inverse was observed for the loamy/sandy soils. This result is mainly associated with the higher molecular weights in the contrasting aggregate sizes (Tables S4 and S5—Supplementary Materials). The same behavior was observed in some loamy/sandy soils when the c. 662 keV photon energy was analyzed.

The atomic (Figure 2e,f and Figure 4e,f) and electronic (Figure 2g,h and Figure 4g,h) cross-sections followed the same behavior of μ/ρ between the aggregate sizes in some of the soils (c. 60 keV) (both soil groups) (Tables S4 and S5—Supplementary Materials) [25,40]. This is related to the σ_A_ and σ_E_ cross-sections’ dependency on μ/ρ (Equations (2) and (3)) and the influence of the total number of molecular formula units for σ_A_ [19,40,41]. These two parameters were also mainly influenced by Fe_2_O_3_ and μ/ρ in the two soil groups and aggregate sizes [16,25,40,42]. For c. 662 keV, the relation between μ/ρ, σ_A_, and σ_E_ was more complex than that observed for c. 60 keV. The Fe_2_O_3_ and SiO_2_ contents explain the results for the two aggregate sizes for c. 662 keV, except the 2–1 mm aggregate sizes in the clayey soils. The highest σ_A_ differences were noticed only in the loamy/sandy soils (Tables S4 and S5—Supplementary Materials). Our results demonstrate that the photoelectric effect, incoherent scattering, and pair production influence the σ_A_ and σ_E_ behavior as a function of the photon energy, similar to μ/ρ [25,38].

The contrasting aggregate sizes influenced Z_eff_ in the clayey soils for c. 60 keV (Tables S4 and S5—Supplementary Materials). The main factors explaining the differences among the soils (c. 60 keV) were the Fe_2_O_3_, μ/ρ, σ_A_, and σ_E_ content [19,43], which presented a direct relation with Z_eff_ in both soil groups (Figure 3a,b and Figure 5a,b). For c. 662 keV (Tables S4 and S5—Supplementary Materials), the results found when comparing the soil groups were mainly related to the contents of SiO_2_, Fe_2_O_3_, μ/ρ, and σ_E_. We must highlight that Z_eff_ is directly associated with the electronic and atomic cross-sections (Equation (4)). This nuclear parameter also depends on Z of the soil elements [17]. The higher Z_eff_ is, the higher the concentration of high Z elements [44]. The decreased Z_eff_ in the energy region from ~10^−2^ to ~10^−1^ MeV is explained by changes in the dominance of the effects responsible for the photon absorption and scattering [17,20]. Similar findings were also found in studies with gemstones [45], granite [46], clay [47], and glasses [40,48].

For N_el_ (Figure 3c,d and Figure 5c,d), our results are directly associated with the relation between this nuclear parameter and Z_eff_ (Equation (5)) [19,25,49]. The curve decays observed from ~10^−2^ to ~10^−1^ MeV are also explained by partial effect dominance changes in the photon absorption and scattering [48]. For c. 60 keV (Tables S4 and S5—Supplementary Materials), N_el_ presented an inverse relation with Fe_2_O_3_, μ/ρ, σ_A_, and σ_E_, explaining the curve inversions compared to Z_eff_. This highlights the influence of these parameters in N_el_, which suffers a direct influence from the SiO_2_ and sand content. For c. 662 keV (Tables S4 and S5—Supplementary Materials), N_el_ did not show any differences between the soil groups and aggregate sizes. The direct and inverse relations between N_el_ and SiO_2_, μ/ρ, sand (direct), and Fe_2_O_3_, σ_E_, and Z_eff_ (inverse) can explain the results obtained among the soils [50].

This study proposes that the size of the soil aggregates influences the radiation interaction parameters. We verified that the major oxides (SiO_2_, Al_2_O_3_, Fe_2_O_3_) found in the tropical/subtropical soils investigated were affected by the aggregate sizes, mainly for the soils containing a high clay content. Consequently, the radiation interaction parameters were also influenced by the aggregate sizes. In general, μ/ρ and Z_eff_ were sensitive to the aggregate sizes in low photon energies (c. 60 keV) in clayey soils. On the other hand, the loamy/sandy soils did not show significant differences in the radiation interaction parameters due to the aggregate sizes. The photon energy increase (c. 662 keV) demonstrated that only Z_eff_ presented the highest differences for most soils. However, we verified that σ_M_, Z_eff_, and N_el_ were the most sensitive parameters to the chemical and granulometric soil compositions. Thus, we observed that although microaggregates can present variations in the soil chemical compositions as a function of their sizes, the distinct compositions found were not enough to induce significant differences in many of the radiation interaction parameters studied. However, our study involved a limited number of soils. In addition, to better analyze the results obtained, a detailed study of the soil mineralogical composition and its fractions is required to explain the differences found in the chemical composition between aggregate sizes [51,52].

Finally, the results presented in our study show that the soil type also influences its radiation interaction parameters as a function of differences in its chemical composition. Since soil is an abundant material that can be used in ionizing radiation attenuation, analysis of how this material affects radiation attenuation becomes relevant. Ionizing radiation can cause severe damage to human health, mainly affecting DNA molecules when exposed to small doses of radiation and leading to death at high doses. Thus, the analysis of alternative materials such as soil to attenuate radiation is necessary to prevent the use of toxic materials such as lead. Thus, the results of our study show how tropical/subtropical soils affect radiation interaction parameters. Furthermore, as soil is a cost-effective material with a diverse composition in tropical countries, its use as a radiation-shielding material can positively impact the preservation of the environment using more sustainable materials.

## 5. Conclusions

Our results showed that the major oxides (SiO_2_, Al_2_O_3_, Fe_2_O_3_) found in the tropical/subtropical soils studied showed variations according to the aggregate sizes (2–1 mm and <45 μm). In the clayey soils, small aggregates showed the most significant amounts of SiO_2_ (majority of soils) and Al_2_O_3_ while Fe_2_O_3_ showed the highest concentrations in the 2–1 mm aggregates. In the loamy/sandy soils, Al_2_O_3_ was found, in general, in large concentrations in small aggregates. SiO_2_ and Fe_2_O_3_ did not present a clear trend between the aggregate sizes. The radiation interaction parameters were influenced by partial effects (photoelectric effect, incoherent scattering, and pair production), mainly explaining the observed variations in the mass attenuation coefficient and atomic and electronic cross-sections as a function of the energy, in both soil groups (clay and loam/sand) and aggregate sizes. The molecular cross-section, effective atomic number, and electron density presented the best discrimination among soils.

When two reference photon energies (c. 60 and c. 662 keV) were considered, we noticed that for the lower energy, the clayey soils showed higher μ/ρ values for aggregates <45 μm, but only three soils showed differences with one another. In the loamy/sandy soils, no differences in μ/ρ were observed between the soils for the different aggregate sizes. Overall, σ_M_ showed higher values for the 2–1 mm aggregates but only in some soils. A similar behavior was observed in the loamy/sandy soils. The parameters σ_A_ and σ_E_ showed differences in some clayey samples but did not differ in the loamy/sandy soils between the aggregate sizes. The Z_eff_ and N_el_ parameters showed higher values in most of the clayey soils for the 2–1 mm aggregates while no difference was observed for these parameters in the loamy/sandy soils. For c. 662 keV, the parameters with the most significant differences between the aggregate sizes for the clayey soils were σ_M_ and Z_eff_. The 2–1 mm aggregates showed the highest values of σ_M_ and Z_eff_ in the clayey soils. Concerning the loamy/sandy soils, μ/ρ, σ_A_, σ_M,_ and Z_eff_ were the parameters with the most remarkable differences between the aggregate sizes.

Thus, we conclude that although the chemical composition of the soil was sensitive to the aggregate size studied, the radiation interaction parameters presented differences for only some of these parameters. Therefore, to obtain better conclusions about the impact of aggregate sizes, we suggest increasing the number of soils studied with different particle size compositions and increasing the range of aggregate sizes to investigate how such aggregates can influence radiation attenuation.

## Figures and Tables

**Table 1:** Soil classification according to its granulometric composition (clay, silt, and sand contents).

Place of Sampling	Sample Acronym	Texture Classification	Clay (%)	Silt (%)	Sand (%)
	Clay				
Rio Azul	RIO	Clay	41	17	42
Ibiporã	IB-1	Clay	47	24	29
Mercedes	MER	Clay	47	29	24
Ibiporã	IB-2	Clay	50	22	28
Ibiporã	IB-3	Clay	59	17	24
Londrina	LDA	Clay	66	16	18
Itaipulândia	ITA	Clay	70	17	13
	Loam/Sand				
Paranavaí	PAR	Sand	8	1	91
Joaquim Távora	JTA	Sandy Loam	18	18	64
Cerro Azul	CAZ	Silt Loam	23	56	21
Lapa	LAP	Sandy Clay Loam	25	21	54
Rio Azul	RAZ	Clay Loam	36	34	30
Morretes	MOR	Clay Loam	38	27	35

**Table 2:** Main oxides measured in the 13 soil types studied with different aggregate sizes (2–1 mm and <45 μm). The average values are followed by the standard deviation, which was rounded according to significant figures.

	Oxides (%)					
Soil	Al_2_O_3_	SiO_2_	Fe_2_O_3_	Al_2_O_3_	SiO_2_	Fe_2_O_3_
	2–1 mm			<45 μm		
	Clay					
RIO	31.23 ± 0.04	52.9 ± 0.4	10.54 ± 0.06	33.9 ± 0.5	55.5 ± 0.1	6.1 ± 0.2
IB-1	33.3 ± 0.4	31.0 ± 0.6	28.8 ± 0.6	36.1 ± 0.4	32.1 ± 0.4	25.8 ± 0.6
MER	30.00 ± 0.06	33.8 ± 0.3	29.2 ± 0.2	31.8 ± 0.7	34 ± 1	27.2 ± 0.2
IB-2	34 ± 1	30.4 ± 0.5	28.6 ± 0.3	37.6 ± 0.5	32.6 ± 0.5	24.36 ± 0.01
IB-3	31.3 ± 0.3	31.3 ± 0.3	30.0 ± 0.6	33.25 ± 0.01	32.9 ± 0.4	27.22 ± 0.11
LDA	37.9 ± 0.2	27.4 ± 0.5	28.1 ± 0.4	40.4 ± 0.1	27.57 ± 0.12	26.55 ± 0.06
ITA	39.7 ± 0.9	32.9 ± 0.3	22.0 ± 0.7	41.4 ± 0.2	31.9 ± 0.1	21.4 ± 0.4
	Loam/Sand					
PAR	27 ± 2	65 ± 3	4.0 ± 0.5	19 ± 1	76 ± 1	3.6 ± 0.1
JTA	21.7 ± 0.5	70 ± 1	3.6 ± 0.4	24.2 ± 0.4	66.7 ± 0.3	4.3 ± 0.2
CAZ	20.5 ± 0.2	69.4 ± 0.4	4.26 ± 0.05	21.21 ± 0.09	71.00 ± 0.05	3.27 ± 0.02
LAP	22.9 ± 0.3	67.3 ± 0.8	4.5 ± 0.2	25.5 ± 0.4	65.8 ± 0.3	4.5 ± 0.2
RAZ	33.9 ± 0.6	55.5 ± 0.9	6.4 ± 0.4	40.35 ± 0.11	47.9 ± 0.2	7.32 ± 0.04
MOR	29.2 ± 0.3	53.6 ± 0.4	11.3 ± 0.1	31.8 ± 0.3	53.8 ± 0.4	9.7 ± 0.2

**Table 3:** Mass attenuation coefficient (μ/ρ) for the photon energies of c. 60 and c. 662 keV of the 13 soil types studied presenting different aggregate sizes (2–1 mm and <45 μm). The average values are followed by the standard deviation, which was rounded according to significant figures.

Soil	Photon Energy (keV)			
	c. 60	c. 662	c. 60	c. 662
	2–1 mm		<45 μm	
	μ/ρ—Clay			
RIO	0.303 ± 0.003	0.0765 ± 0.0001	0.308 ± 0.002	0.0765 ± 0.0001
IB-1	0.462 ± 0.005	0.0760 ± 0.0001	0.50 ± 0.03	0.0760 ± 0.0001
MER	0.48 ± 0.01	0.0760 ± 0.0001	0.52 ± 0.04	0.0760 ± 0.0001
IB-2	0.459 ± 0.003	0.0760 ± 0.0001	0.47 ± 0.03	0.0760 ± 0.0001
IB-3	0.48 ± 0.01	0.0760 ± 0.0001	0.51 ± 0.06	0.0760 ± 0.0001
LDA	0.463 ± 0.011	0.0759 ± 0.0001	0.514 ± 0.005	0.0759 ± 0.0001
I-02-0	0.417 ± 0.006	0.0760 ± 0.0001	0.469 ± 0.001	0.0760 ± 0.0001
	μ/ρ—Loam/Sand			
PAR	0.283 ± 0.003	0.0768 ± 0.0001	0.282 ± 0.005	0.0769 ± 0.0001
JTA	0.291 ± 0.009	0.0768 ± 0.0001	0.283 ± 0.008	0.0768 ± 0.0001
CAZ	0.298 ± 0.008	0.0768 ± 0.0001	0.288 ± 0.006	0.0768 ± 0.0001
LAP	0.292 ± 0.001	0.0768 ± 0.0001	0.294 ± 0.006	0.0768 ± 0.0001
RAZ	0.296 ± 0.008	0.0767 ± 0.0001	0.288 ± 0.001	0.0767 ± 0.0001
MOR	0.340 ± 0.001	0.0765 ± 0.0001	0.34 ± 0.02	0.0765 ± 0.0001

## Data Availability

Available upon request.

## Supplementary Materials

The following supporting information can be downloaded at https://www.mdpi.com/article/10.3390/ijerph192214635/s1: Figure S1: Texture triangle (USDA) presenting the distribution of the soil types studied; Table S1: Main oxides measured in the 13 soil types studied for the 2–1 mm aggregate size. The values between parentheses represent the standard deviation. The average values and standard deviation were rounded according to significant figures; Table S2: Main oxides measured in the 13 soil types studied for the <45 μm aggregate size. The values between parentheses represent the standard deviation. The average values and standard deviation were rounded according to significant figures; Table S3: Experimental (EXP) and calculated (XCOM) mass attenuation coefficients in the 13 soil types studied. The relative change was obtained considering the experimental method as a reference ($RG=\left|\frac{y_{XCOM}-y_{EXP}}{y_{EXP}}\right|\times 100$). For the RG analysis, the aggregate size between 2 and 1 mm was selected. * Aggregates with sizes between 2 and 1 mm. ** Aggregates with sizes <45 μm; Table S4: Molecular (σ_M_), atomic (σ_A_), electronic (σ_E_) cross-sections, effective atomic number (Z_eff_), and electron density (N_el_) of the 13 soil types studied for the 2–1 mm aggregate sizes. The values between parentheses represent the standard deviation. The average values and standard deviation were rounded according to significant figures; Table S5: Molecular (σ_M_), atomic (σ_A_), electronic (σ_E_) cross-sections, effective atomic number (Z_eff_), and electron density (N_el_) of the 13 soil types studied for the <45 μm aggregate sizes. The values between parentheses represent the standard deviation. The average values and standard deviation were rounded according to significant figures.
