# Supplementary material for: Insights into the Effect of Aggregate Sizes on the Soil Radiation Interaction Properties Based on X-ray Fluorescence
Source: Int J Environ Res Public Health. 2022 Nov 8;19(22):14635. doi: 10.3390/ijerph192214635 (PMC9690324; doi:10.3390/ijerph192214635)
Supplement: Supplementary file 1 [file ijerph-19-14635-s001.zip › ijerph-1920351-supplementary.pdf]

Insights into the effect of aggregate sizes on the soil radiation interaction properties based on X-ray fluorescence

SUPPLEMENTARY MATERIAL

The studied soils showed a wide range of textures: clay, silt-loam, sand-loam, sand-clay-loam, sand, and clay-loam. The following textural triangle presents the distribution of the different analyzed soil textures (Figure S1).

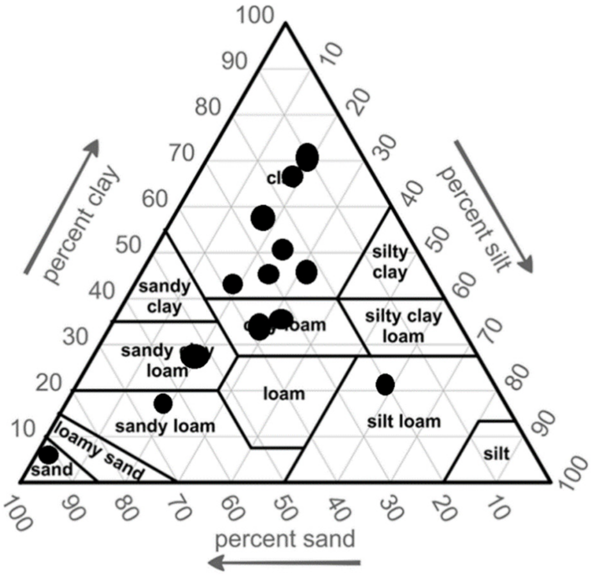

Figure S1. Texture triangle (USDA) presenting the distribution of the soil types studied.

The main oxides found in the 13 soils studied are presented in Tables S1 and S2.

Table S1. Main oxides measured in the 13 soil types studied for the 2-1 mm aggregate size. The values between parentheses represent the standard deviation. The average values and standard deviation were rounded according to significant figures.

	2-1 mm					
	Oxide (%)					
	Al_2O_3	SiO_2	Fe_2O_3	K_2O	TiO_2	SO_3
**Soil**						
	**Clay**					
RIO	33.94<br>(0.04)	55.6<br>(0.4)	6.41<br>(0.06)	-	1.71<br>(0.01)	1.34<br>(0.12)
IB-1	34.1<br>(0.4)	31.0<br>(0.6)	28.8<br>(0.6)	-	4.37<br>(0.05)	1.24<br>(0.11)
MER	30.00<br>(0.06)	33.8<br>(0.3)	29.2<br>(0.2)	-	4.87<br>(0.03)	1.3<br>(0.3)
IB-2	40<br>(1)	30.4<br>(0.5)	28.6<br>(0.3)	-	3.3<br>(0.1)	1.5<br>(0.2)

IB-3	31.3	31.3	30.0	-	3.9	1.4
	(0.3)	(0.3)	(0.6)		(0.2)	(0.3)
LDA	38.0	27.4	28.1	-	3.64	1.7
	(0.2)	(0.5)	(0.4)		(0.02)	(0.3)
ITA	40.0	33.0	22.0	-	3.19	1.21
	(0.9)	(0.3)	(0.7)		(0.11)	(0.12)
			**Loam/Sand**			
PAR	27	65	4	-	1.2	1.9
	(3)	(3)	(1)		(0.1)	(0.2)
JTA	21.7	70	3.6	1.91	0.75	1.8
	(0.5)	(1)	(0.4)	(0.03)	(0.06)	(0.4)
CAZ	20.5	69.4	4.26	2.07	1.06	2.2
	(0.2)	(0.4)	(0.05)	(0.02)	(0.01)	(0.3)
LAP	22.9	67.3	5.5	1.32	0.99	2.4
	(0.3)	(0.8)	(0.2)	(0.01)	(0.04)	(0.3)
RAZ	26.8	63.3	4.6	1.95	0.75	2.2
	(0.3)	(0.9)	(0.3)	(0.03)	(0.02)	(0.2)
MOR	29.2	53.6	11.3	-	2.92	2.0
	(0.3)	(0.4)	(0.1)		(0.08)	(0.2)

Table S2. Main oxides measured in the 13 soil types studied for the <45 μm aggregate size. The values between parentheses represent the standard deviation. The average values and standard deviation were rounded according to significant figures.

				**<45 μm**			
				**Oxide (%)**			
	**Al_2O_3**	**SiO_2**	**Fe_2O_3**	**K_2O**	**TiO_2**	**BaO**	**SO_3**
**Soil**							
				**Clay**			
RIO	40.8	31.5	22.1	-	3.3	-	1.3
	(0.5)	(0.1)	(0.2)		(0.2)		(0.2)
IB-2	36.1	32.1	25.8	-	3.1	-	1.13
	(0.4)	(0.4)	(0.6)		(0.2)		(0.01)
MER	31.8	34	27.2	-	3.9	-	1.2
	(0.7)	(1)	(0.2)		(0.3)		(0.2)
IB-2	37.6	32.6	24.36	-	2.4	-	1.1
	(0.5)	(0.5)	(0.01)		(0.3)		(0.1)
IB-3	33.25	32.9	27.22	-	3.5	0.39	0.78
	(0.01)	(0.4)	(0.11)		(0.4)	(0.01)	(0.05)
LDA	40.4	27.57	26.55	-	2.94	-	1.0
	(0.1)	(0.12)	(0.06)		(0.02)		(0.2)
ITA	41.4	31.9	21.4	-	2.61	0.98	1.22
	(0.2)	(0.1)	(0.4)		(0.06)	(0.03)	(0.03)
				**Loam/Sand**			
PAR	19	76	3.56	-	0.68	-	1.28
	(1)	(1)	(0.08)		(0.01)		(0.05)
JTA	24.2	66.7	4.3	2.03	0.82	-	1.2
	(0.4)	(0.3)	(0.1)	(0.03)	(0.05)		(0.1)
CAZ	21.21	71.00	3.27	1.89	0.83	-	1.42
	(0.09)	(0.05)	(0.02)	(0.01)	(0.03)		(0.09)

LAP	25.5<br>(0.4)	65.8<br>(0.3)	4.5<br>(0.2)	1.37<br>(0.03)	0.91<br>(0.01)	-	1.56<br>(0.02)
RAZ	30.6<br>(0.1)	60.9<br>(0.2)	4.29<br>(0.03)	1.97<br>(0.02)	0.75<br>(0.01)	-	1.25<br>(0.05)
MOR	31.8<br>(0.3)	53.8<br>(0.4)	9.7<br>(0.2)	-	2.35<br>(0.07)	-	1.37<br>(0.09)

Table S3 shows the experimental and theoretical attenuation coefficient results for the 13 soils studied. For the experimental measurement of the mass attenuation coefficient, first the linear attenuation coefficient was calculated through the Beer-Lambert equation:

$$\mu = \frac{1}{x} ln\left(\frac{I_0}{I}\right) \qquad (6)$$

where I is the intensity after transmission through the sample of thickness x and I_0 is the incident photon intensity (no sample), respectively. The soil density (ρ) was obtained through the relation between the dry soil mass and the internal volume of the acrylic container filled with soil. The mass attenuation coefficient was then obtained using information about the density of the soils.

Two radioactive sources were employed for the experimental measurements: ^{241}Am and ^{137}Cs. These two sources produce gamma-ray photons with energies of 59.5 keV (^{241}Am) and 661.6 keV (^{137}Cs). The photons were detected using a NaI(Tl) solid scintillation detector of flat type. This detector type was chosen due to its high efficiency. The detector was coupled to standard spectrometric gamma electronics allowing us to control the photomultiplier tube high-voltage, select the photopeak, adjust the time of measurement, and count the photons detected.

For the experimental measurements, appropriate geometry was achieved using circular lead collimators adjusted in front of the radioactive source (2 mm) and in front of the detector entrance (4.5 mm). The distance between the source and the detector was 23 cm aligned using a laser pointer. For the experimental evaluation of μ, disturbed soil samples were dried in a forced air circulation oven (105 °C for 24 h) and sieved in a 1 mm mesh sieve. After that, samples were gently packed into a thin wall (0.5 cm) acrylic container (10 × 10 × 10 cm). The time interval selected for each measurement was 600 s, which allowed us to obtain deviations smaller than 0.2% (^{137}Cs) and 0.7% (^{241}Am) due to the statistical uncertainty of the radioactive source's gamma-ray photon emissions.

Table S3. Experimental (EXP) and calculated (XCOM) mass attenuation coefficients (μ/ρ) for the 13 soil types studied. The relative change was obtained considering the experimental method as a reference ($RG = \left|\frac{y_{XCOM}-y_{EXP}}{y_{EXP}}\right| \times 100$). For the RG analysis, the aggregate size between 2 and 1 mm was selected. * Aggregates with sizes between 2 and 1 mm. ** Aggregates with sizes <45 μm.

Soil	Photon energy (keV)							
	EXP		XCOM				RG (%)	RG (%)
	c. 60	c. 662	c. 60*	c. 662*	c. 60**	c. 662**	2-1 mm	
			μ/ρ (cm² g⁻¹) – Clay					
RIO	0.290	0.0733	0.303	0.0765	0.308	0.0765	4.5	4.4
IB-1	0.467	0.0754	0.462	0.0760	0.498	0.0760	1.1	0.8
MER	0.471	0.0768	0.481	0.0760	0.520	0.0760	2.1	1.0
IB-2	0.436	0.0777	0.459	0.0760	0.468	0.0760	5.3	2.2
IB-3	0.453	0.0779	0.482	0.0760	0.509	0.0760	6.4	2.4
LDA	0.436	0.0748	0.463	0.0759	0.514	0.0759	6.2	1.5
ITA	0.398	0.0780	0.417	0.0760	0.469	0.0760	4.8	2.6
			μ/ρ (cm² g⁻¹) – Loam/Sand					
PAR	0.266	0.0783	0.283	0.0768	0.282	0.0769	6.4	1.9
JTA	0.279	0.0777	0.291	0.0768	0.283	0.0768	4.3	1.2
CAZ	0.278	0.0785	0.298	0.0768	0.288	0.0768	7.2	2.2
LAP	0.280	0.0772	0.292	0.0768	0.294	0.0768	4.3	0.5
RAZ	0.290	0.0782	0.296	0.0767	0.288	0.0767	2.1	1.9
MOR	0.318	0.0769	0.340	0.0765	0.336	0.0765	6.9	0.5

Table S4 in the sequence brings the radiation interaction parameters for the c. 60 keV and c. 662 keV photon energies for the clayey and loamy/sandy soils for the 2-1 mm aggregate sizes.

Table S4. Molecular (σ_M), atomic (σ_A), electronic (σ_E) cross-sections, effective atomic number (Z_{eff}) and electron density (N_{el}) of the 13 soil types studied for the 2-1 mm aggregate sizes. The values between parentheses represent the standard deviation. The average values and standard deviation were rounded according to significant figures.

2-1 mm					
Parameter	σ_M	σ_A	σ_E	Z_{eff}	N_{el}
	(barn/molecule)	(barn/atom)	(barn/electron)		($\times 10^{23}$ electrons g⁻¹)
			c. 60 keV		
Soil					
			Clay		
RIO	1.9×10^{6}<br>(0.6×10^{6})	12<br>(1)	1.03<br>(0.01)	10.20<br>(0.01)	2.92<br>(0.01)
IB-1	2.94×10^{5}<br>(0.09×10^{5})	17.7<br>(0.3)	1.62<br>(0.02)	10.94<br>(0.04)	2.86<br>(0.01)
MER	1.25×10^{6}<br>(0.07×10^{6})	18.5<br>(0.6)	1.69<br>(0.06)	10.97<br>(0.08)	2.85<br>(0.02)
IB-2	2.00×10^{6}<br>(0.09×10^{6})	17.5<br>(0.1)	1.60<br>(0.01)	10.92<br>(0.01)	2.86<br>(0.01)

IB-3	1.8×10^{6} (0.7×10^{6})	18.6 (0.6)	1.69 (0.06)	11.03 (0.01)	2.86 (0.01)
LDA	3.4×10^{5} (0.4×10^{5})	17.7 (0.4)	1.63 (0.04)	10.84 (0.05)	2.84 (0.01)
ITA	$4x10^{5}$ (2×10^{5})	15.5 (0.2)	1.46 (0.03)	10.65 (0.08)	2.86 (0.01)
**Loam/Sand**					
PAR	3×10^{5} (2×10^{5})	9.7 (0.2)	0.96 (0.01)	10.09 (0.01)	2.95 (0.01)
JTA	2×10^{6} (1×10^{6})	10.0 (0.3)	0.99 (0.05)	10.14 (0.03)	2.92 (0.05)
CAZ	2.6×10^{6} (0.3×10^{6})	10.3 (0.3)	1.00 (0.04)	10.09 (0.04)	2.92 (0.02)
LAP	1.7×10^{6} (0.6×10^{6})	10.06 (0.06)	0.993 (0.01)	10.13 (0.01)	2.94 (0.01)
RAZ	1.9×10^{6} (0.5×10^{6})	10.2 (0.3)	1.00 (0.02)	10.14 (0.03)	2.94 (0.01)
MOR	9×10^{5} (2×10^{5})	12.06 (0.06)	1.19 (0.03)	10.31 (0.01)	2.91 (0.01)
**c. 662 keV**					
**Clay**					
RIO	5×10^{5} (2×10^{5})	2.9 (0.3)	0.26 (0.01)	10.28 (0.01)	2.96 (0.01)
IB-1	4.8×10^{4} (0.2×10^{4})	2.91 (0.01)	0.26 (0.01)	11.22 (0.04)	2.93 (0.01)
MER	1.9×10^{5} (0.6×10^{5})	2.92 (0.01)	0.26 (0.01)	11.28 (0.02)	2.93 (0.01)
IB-2	3.3×10^{5} (0.2×10^{5})	2.90 (0.01)	0.26 (0.01)	11.20 (0.02)	2.93 (0.01)
IB-3	2.9×10^{5} (1.1×10^{5})	2.97 (0.06)	0.26 (0.01)	11.29 (0.03)	2.93 (0.01)
LDA	5.6×10^{4} (0.6×10^{4})	2.89 (0.01)	0.26 (0.01)	11.15 (0.01)	2.92 (0.01)
ITA	7×10^{4} (3×10^{4})	2.82 (0.01)	0.26 (0.01)	10.92 (0.06)	2.94 (0.01)
**Loam/Sand**					
PAR	9×10^{4} (5×10^{4})	2.63 (0.01)	0.26 (0.01)	10.18 (0.02)	2.97 (0.01)
JTA	6×10^{5} (4×10^{5})	2.64 (0.01)	0.26 (0.01)	10.20 (0.01)	2.97 (0.01)
CAZ	6.7×10^{5} (0.6×10^{5})	2.65 (0.01)	0.26 (0.01)	10.26 (0.01)	2.97 (0.01)
LAP	5×10^{5} (2×10^{5})	2.65 (0.01)	0.26 (0.01)	10.24 (0.01)	2.97 (0.01)
RAZ	5×10^{5} (1×10^{5})	2.65 (0.01)	0.26 (0.01)	10.23 (0.02)	2.97 (0.01)
MOR	2.1×10^{5} (0.4×10^{5})	2.71 (0.01)	0.26 (0.01)	10.47 (0.01)	2.96 (0.01)

Table S5 in the sequence brings the radiation interaction parameters for the c. 60 keV and c. 662 keV photon energies for the clayey and loamy/sandy soils for the <45 μm aggregate sizes.

Table S5. Molecular (σ_M), atomic (σ_A), electronic (σ_E) cross-sections, effective atomic number (Z_{eff}), and electron density (N_{el}) of the 13 soil types studied for the <45 μm aggregate sizes. The values between parentheses represent the standard deviation. The average values and standard deviation were rounded according to significant figures.

		<45 μm			
**Parameter**	**σ_M**	**σ_A**	**σ_E**	**Z_{eff}**	**N_{el}**
	**(barn/molecule)**	**(barn/atom)**	**(barn/electron)**		**($\times10^{23}$ electrons g^{-1})**
		**c. 60 keV**			
**Soil**					
		**Clay**			
RIO	2.0×10^6	10.74	1.05	10.21	2.93
	(0.2×10^6)	(0.02)	(0.01)	(0.02)	(0.01)
IB-1	3×10^5	19	1.80	10.64	2.80
	(1×10^5)	(1)	(0.12)	(0.04)	(0.02)
MER	3.7×10^5	20	1.9	10.72	2.80
	(0.5×10^5)	(2)	(0.2)	(0.11)	(0.04)
IB-2	3.8×10^5	18	1.66	10.64	2.83
	(0.1×10^5)	(1)	(0.11)	(0.11)	(0.04)
IB-3	1.4×10^6	19	1.8	10.8	2.82
	(0.5×10^6)	(2)	(0.2)	(0.2)	(0.05)
LDA	4.0×10^5	19.6	1.78	10.62	2.79
	(0.2×10^5)	(0.2)	(0.08)	(0.01)	(0.01)
ITA	1.81×10^5	17.45	1.67	10.43	2.80
	(0.04×10^5)	(0.03)	(0.01)	(0.02)	(0.01)
		**Loam/Sand**			
PAR	4×10^5	9.6	0.95	10.05	2.95
	(3×10^5)	(0.2)	(0.02)	(0.03)	(0.01)
JTA	2.85×10^6	10.5	1.04	10.10	2.92
	(0.08×10^6)	(0.3)	(0.04)	(0.05)	(0.02)
CAZ	3×10^6	9.9	0.98	10.11	2.95
	(1×10^6)	(0.2)	(0.02)	(0.04)	(0.01)
LAP	3.4×10^6	10.1	1.07	10.11	2.94
	(0.4×10^6)	(0.2)	(0.12)	(0.03)	(0.01)
RAZ	3.0×10^6	9.93	0.99	10.14	2.94
	(0.9×10^6)	(0.01)	(0.02)	(0.01)	(0.01)
MOR	1.4×10^6	11.42	1.19	10.19	2.90
	(0.1×10^6)	(0.01)	(0.12)	(0.06)	(0.02)
		**c. 662 keV**			
		**Clay**			
RIO	5.2×10^5	0.26	2.58	10.33	2.96
	(0.4×10^5)	(0.02)	(0.01)	(0.02)	(0.01)
IB-1	5×10^4	0.26	2.60	11.11	2.93
	(1×10^4)	(0.01)	(0.01)	(0.04)	(0.01)
MER	5.4×10^4	0.26	2.60	11.19	2.92

	(0.3×10^4)	(0.01)	(0.01)	(0.05)	(0.01)
IB-2	6.2×10^4	0.26	2.59	11.04	2.94
	(0.2×10^4)	(0.01)	(0.01)	(0.01)	(0.01)
IB-3	1.9×10^5	0.26	2.61	11.14	2.91
	(0.7×10^5)	(0.01)	(0.01)	(0.04)	(0.02)
LDA	6.0×10^4	0.26	2.59	11.13	2.92
	(0.3×10^4)	(0.01)	(0.01)	(0.01)	(0.01)
ITA	2.94×10^4	0.26	2.59	10.91	2.93
	(0.08×10^4)	(0.01)	(0.01)	(0.01)	(0.01)
			**Loam/Sand**		
PAR	1.2×10^5	0.26	2.58	10.14	2.98
	(0.7×10^5)	(0.01)	(0.01)	(0.01)	(0.01)
JTA	7.2×10^5	0.26	2.58	10.28	2.97
	(0.4×10^5)	(0.01)	(0.01)	(0.01)	(0.01)
CA	7×10^5	0.26	2.58	10.20	2.98
	(3×10^5)	(0.01)	(0.01)	(0.01)	(0.01)
LAP	9×10^5	0.26	2.58	10.24	2.97
	(1×10^5)	(0.01)	(0.01)	(0.01)	(0.01)
RAZ	8×10^5	0.26	2.58	10.23	2.97
	(2×10^5)	(0.01)	(0.01)	(0.01)	(0.01)
MOR	3.1×10^5	0.26	2.58	10.41	2.96
	(0.2×10^5)	(0.01)	(0.01)	(0.02)	(0.01)
